# Structural Basis for Allosteric Ligand Recognition in the Human CC Chemokine Receptor 7

**DOI:** 10.1016/j.cell.2019.07.028

**Published:** 2019-08-22

**Authors:** Kathrin Jaeger, Steffen Bruenle, Tobias Weinert, Wolfgang Guba, Jonas Muehle, Takuya Miyazaki, Martin Weber, Antonia Furrer, Noemi Haenggi, Tim Tetaz, Chia-Ying Huang, Daniel Mattle, Jean-Marie Vonach, Alain Gast, Andreas Kuglstatter, Markus G. Rudolph, Przemyslaw Nogly, Joerg Benz, Roger J.P. Dawson, Joerg Standfuss

**Affiliations:** 1 Laboratory of Biomolecular Research, Department of Biology and Chemistry, Paul Scherrer Institute, Villigen PSI; 2 Roche Pharma Research and Early Development, Therapeutic Modalities, Roche Innovation Center Basel, F. Hoffmann-La Roche Ltd, 4070 Basel, Switzerland; 3 Chugai Pharmaceutical Co., Ltd., Research Division, Kamakura Research Labs, Kamakura, Kanagawa, Japan; 4 Macromolecular Crystallography, Swiss Light Source, Paul Scherrer Institute, 5232 Villigen PSI, Switzerland

**Keywords:** chemokine receptors, CCR7, crystal structure, G protein-coupled receptors, allosteric modulation, structure-based drug screening, lymph node metastasis, cancer, membrane proteins

## Abstract

The CC chemokine receptor 7 (CCR7) balances immunity and tolerance by homeostatic trafficking of immune cells. In cancer, CCR7-mediated trafficking leads to lymph node metastasis, suggesting the receptor as a promising therapeutic target. Here, we present the crystal structure of human CCR7 fused to the protein Sialidase NanA by using data up to 2.1 Å resolution. The structure shows the ligand Cmp2105 bound to an intracellular allosteric binding pocket. A sulfonamide group, characteristic for various chemokine receptor ligands, binds to a patch of conserved residues in the Gi protein binding region between transmembrane helix 7 and helix 8. We demonstrate how structural data can be used in combination with a compound repository and automated thermal stability screening to identify and modulate allosteric chemokine receptor antagonists. We detect both novel (CS-1 and CS-2) and clinically relevant (CXCR1-CXCR2 phase-II antagonist Navarixin) CCR7 modulators with implications for multi-target strategies against cancer.

## Highlights

• Crystal structure of the CC chemokine receptor 7
• Cmp2105 is an intracellular allosteric CCR7 receptor antagonist
• The TM7-H8 turn is a pharmacological hotspot for targeting chemokine receptors
• Navarixin is a multi-target antagonist with implications for cancer therapy

## Graphical Abstract

**Figure:**
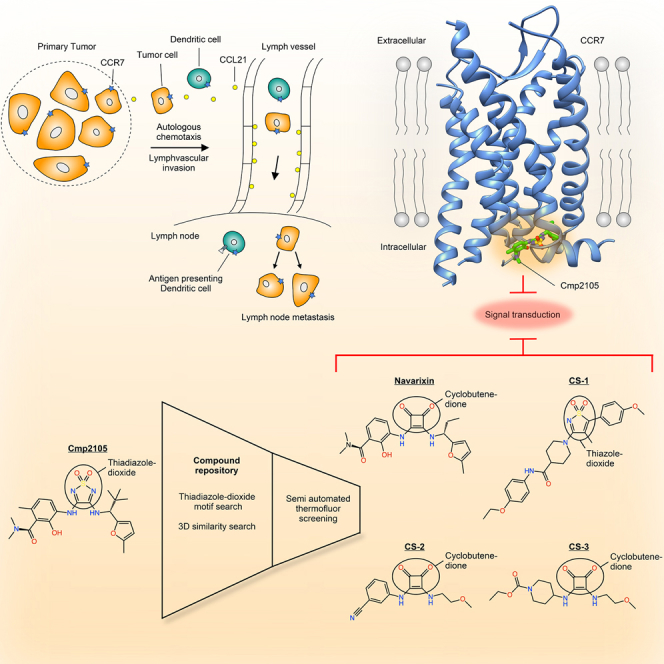

## Teaser

An engineered version of human CCR7, fused to Sialidase NanA, is used to overcome a protein crystallization challenge, leading to a high-resolution structure of CCR7 bound to an intracellular domain antagonist and a computational screen that identifies a series of CCR7 modulators, including Navarixin.

## Introduction

A key feature of the human immune system is the ability to protect against pathogens without targeting healthy cells or tissues. Chemotactic trafficking of the immune response is orchestrated by 20 G protein-coupled receptors (GPCRs) and over 40 chemokines that guide patrolling immune cells to the right place at the right time. Inflammatory chemokines and their receptors are induced upon inflammatory stimuli, whereas homeostatic chemokines are expressed constitutively and create “cellular highways” that constantly navigate cells to specific organs. Important homeostatic CC motif chemokine ligands are CCL19 and CCL21, which bind the CC chemokine receptor 7 (CCR7) to guide B cells, T cells, and antigen-presenting dendritic cells to lymph nodes throughout the body (Förster et al., 1999, Förster et al., 2008).

CCR7 and its ligands are essential components in an autoimmune model of rheumatoid arthritis (Moschovakis et al., 2018). Moreover, pathogenic bacteria can use CCR7-mediated migration of dendritic cells to transfer to draining lymph nodes, from where they can spread to other organs (Förster et al., 2008, Pron et al., 2001). CCR7 is further associated with a wide variety of cancers (Balkwill, 2012, Günther et al., 2005, Müller et al., 2001, Shields et al., 2007), where chemotactic trafficking allows cancer cells to spread by lymph node metastasis (Cunningham et al., 2010, Wiley et al., 2001, Zlotnik et al., 2011). CCR7 expression in colorectal carcinoma, the second most common malignant tumor worldwide (Ferlay et al., 2015), is linked to lymphovascular invasion and decreased survival rates (Günther et al., 2005). Small molecule ligands designed to silence CCR7 thus have great potential to address lymph node metastasis, a major cause for cancer-associated mortality.

So far it has been difficult to find a suitable variety of small molecule ligands as starting points to develop CCR7-targeting drugs. Furthermore, other chemokine receptors, including CXCR1, CXCR2, CCR5, and CXCR4, have also been implicated with cancer metastasis to specific organs (Balkwill, 2012, Mishan et al., 2016, Zlotnik et al., 2011) and thus might compensate for each other if only one of them is targeted. On the other hand, small molecules need to have a certain level of selectivity to minimize side effects during treatment. Structural information is needed to develop drugs that can fulfill these potentially conflicting requirements and much effort has been devoted to determining the structures of chemokine receptors in complex with either synthetic ligands (Oswald et al., 2016, Tan et al., 2013, Wu et al., 2010, Zheng et al., 2016) or native chemokines (Burg et al., 2015, Qin et al., 2015).

Despite several clinical trials, only a few drugs have been approved for the large and highly polar orthosteric chemokine binding pocket (Horuk, 2009, Solari et al., 2015). Spatially distinct allosteric sites in chemokine receptors (Oswald et al., 2016, Zheng et al., 2016) and other GPCRs (Thal et al., 2018) are more compact and promise higher sub-type selectivity than orthosteric sites and are thus widely discussed as exciting possibilities in modern drug discovery (Hauser et al., 2017). Here, we present the crystal structure of CCR7 bound to the allosteric antagonist Cmp2105. This small molecule contains a thiadiazole-dioxide scaffold, which was developed and patented as a potent motif to target CXC- and CC-chemokine receptors (Taveras et al., 2010). We conclude from new ligands found by 3D shape similarity screenings and verified by automated thermofluor screening (Mattle et al., 2018) that thiadiazole-dioxide, thiazole-dioxide, or cyclobutene-dione motifs are key for interacting with a conserved transmembrane helix 7-helix 8 (TM7-H8) protein patch in the Gi protein binding site in CCR7 and other chemokine receptors. Our results suggest this conserved motif as a promising hotspot for targeting chemokine receptors with pharmaceuticals.

### Structure Determination

**Figure S1:**
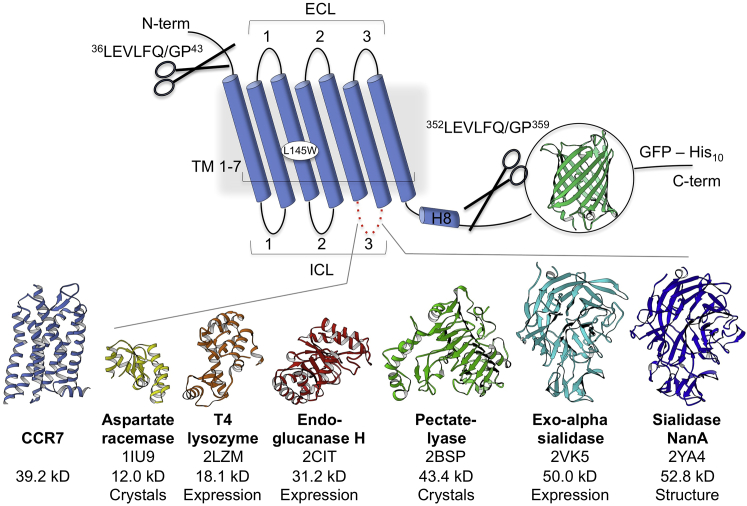
Related to Star Methods Section CCR7 Constructs and Expression Schematic illustration of constructs for CCR7 crystallization screening. Six rounds of construct design and screening were carried out. Expression constructs contained a C-terminal fusion of enhanced green fluorescent protein (eGFP) (Cormack et al., 1996) to determine expression levels followed by a decahistidine-tag (His10) for affinity chromatography purification. Human Rhinovirus 3C protease (Cordingley et al., 1990) recognition sequences were introduced to cleave the N- and C terminus during purification. A replacement of leucine residue at position 145 with tryptophan (Roth et al., 2008) improved thermal stability of the receptor. A series of 17 soluble proteins were selected from the protein data bank for insertion into intracellular loop 3 based on following selection criteria: (1) virtually parallel N- and C-termini with a distance between 6–15 Å. (2) structures available at a resolution better than 2.0 Å. (3) no disulfide bridges or posttranslational modifications. (4) A maximum number of 600 inserted residues v) final manual verification to ensure a maximal diversity of protein folds. Six fusion proteins (shown with name, PDB code, and molecular weight) were chosen for large scale expression of which three crystallized. The Sialidase NanA fusion yielded the best diffracting crystals and was chosen for a final round of optimization resulting in the structure of the CCR7-Sialidase NanA fusion protein.

**Figure 1:**
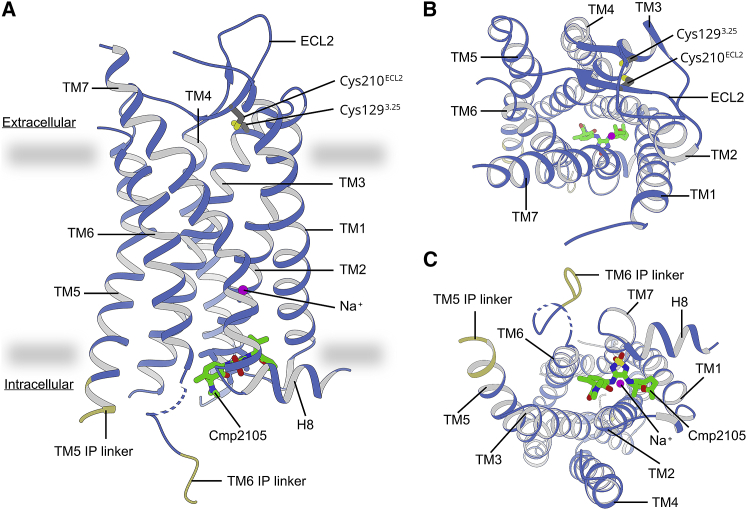
Structure of CCR7 Bound to Cmp2105 The human CCR7 shares the seven transmembrane (TM1–TM7) helical fold of GPCRs where helices are connected by three intracellular (ICL) and three extracellular (ECL) loops. A short aliphatic helix (H8) anchors CCR7 in the cytoplasmic side of the membrane. CCR7 (light blue) viewed in parallel to (A) and from the extracellular (B) and intracellular (C) sides of the membrane (indicated by gray boundaries). The connecting linkers to the fusion protein are labeled (yellow) with two unresolved residues indicated as dashed lines. The small molecule antagonist Cmp2105 (green sticks) binds at the intracellular side of CCR7, similar as described for binding of Cmpd-15PA to the β2-adrenergic receptor, binding of CCR2-RA-[*R*] to CCR2 and binding of Vercirnon to CCR9.

**Figure S2:**
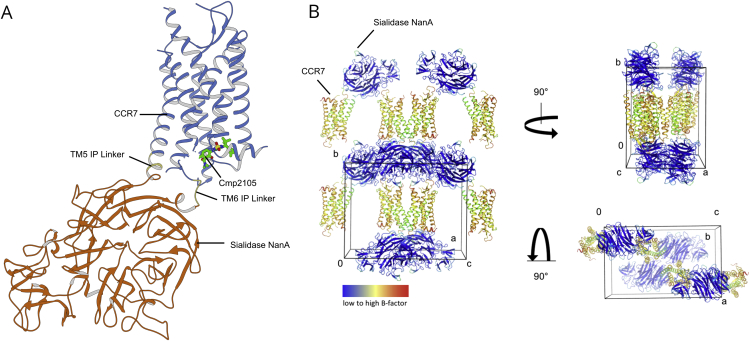
Related to Figure 1 (A) CCR7-Sialidase NanA crystal structure with allosteric antagonist Cmp2105. The chemokine receptor CCR7 (blue) is connected by two linkers in TM5 and TM6 (yellow) to the fusion protein Sialidase NanA (orange) which is located 9.2 Å apart from Cmp2105. (B) Crystal lattice arrangement viewed from three different angles. Crystal contacts exist between adjacent Sialidase NanA and the CCR7 loops ECL1 and ECL2. B-factors (red to blue) are higher for the receptor domain (mean B-factor: 100.6 Å^2^) than for the fusion protein (mean B-factor: 21.7 Å^2^). The B-factors indicate a greater flexibility of the receptor compared to the fusion protein Sialidase NanA. Because of the few crystal contacts restraining the receptor, future studies on conformational dynamics at room temperature might be feasible. The large size of the fusion protein may further qualify the CCR7-Sialidase NanA construct for single particle analysis using electron microscopy.

**Figure S3:**
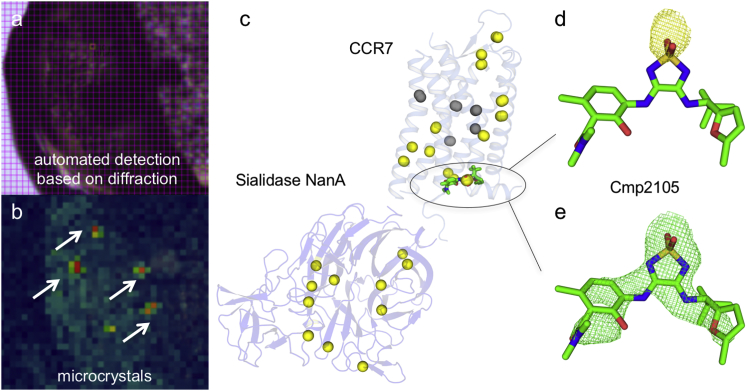
Related to Figure 2 Automated serial data collection was performed to identify the location of native sulfur atoms and assist model building of CCR7. Single-wavelength anomalous dispersion of sulfur atoms (S-SAD) is a routine phasing method (Weinert et al., 2015) that does not need derivatization of the target protein. However, the method is challenging in cases where only weakly diffracting microcrystals are available because it requires very accurate measurements of anomalous differences vulnerable to radiation damage. Automated serial data collection based on raster scanning of mounted mesh loops and automated selection of crystals using X-ray diffraction at 6 keV (A) before and (B) after diffraction screening, blue to red indicates diffraction power at a 5x5 μm^2^ grid point) provides a solution to this problem (Wojdyla et al., 2018, Wojdyla et al., 2016) as the required dose can be spread over small wedges of data collected from thousands of crystals with sizes of only a few micrometers. Diffraction data was collected on 2343 crystals within 24 h of beamtime. Data from the best 726 crystals were combined into a high multiplicity dataset (Table S1). The anomalous signal in these data provided the position of sulfur atoms in the CCR7-Sialidase NanA fusion protein (C) (yellow spheres indicate positions with signal above and gray below 3.0 σ) and Cmp2105 (D) (yellow mesh, 3.0 σ). Additional phase information was further used to assist structure determination by molecular replacement. The location of Cmp2105 was confirmed by simulated annealing F_obs_-F_calc_ omit maps (E) (green mesh, 2.5 σ).

**Figure S4:**
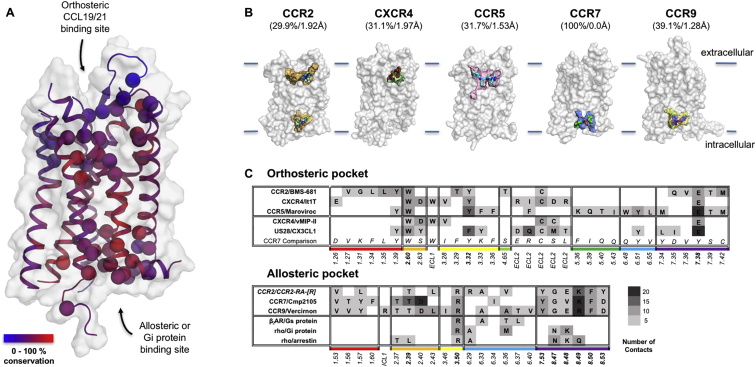
Conservation Patterns of the CCR7 Orthosteric and Allosteric Ligand Binding Site Compared to Other Chemokine Receptors, Related to Figure 3 (A) Small molecules can inhibit chemokine receptors through either the extracellular orthosteric binding pocket, the chemokine (e.g., CCL19/21) activation site, or the intracellular allosteric pocket, which interacts with signaling partners like G proteins. The Cα backbone atoms of residues in contact with small molecule ligands in human chemokine receptors are plotted on the structure of CCR7 (spheres, conservation from blue to red). (B) Illustration of the size, shape, and position of the small molecule binding pockets in respect to the cellular membrane (blue bars). Numbers in brackets indicate sequence identity to CCR7 and root means square deviation of Cα atoms in transmembrane helices. (C) Interatomic contacts (defined as pair of atoms with < 4 Å distance) between chemokine receptors with small molecule ligands (PDB: 5T1A, 3ODU, 4MBS, and 5LWE), chemokines (PDB: 4RWS and 4XT1) and a selection of GPCR-effector complexes (PDB: 3SN6, 6CMO, and 5DGY). Overall a higher overlap between contact sites is observed in the tight intracellular allosteric binding pocket overlapping with the GPCR-effector binding site. The gray-scale indicates the number of contacts to the respective ligand. The orthosteric binding pocket has a higher level of sequence variation compared to the allosteric site. It is wide open and highly polar to allow binding of chemokines which are, with approximate sizes of 8 to 12 kDa, much larger compared to a typical class A GPCR ligand. Synthetic small molecule ligands of chemokine receptors, therefore, target a smaller subpocket as observed for IT1t in CXCR4 (Wu et al., 2010) and BMS-681 in CCR2 (Zheng et al., 2016) or several subpockets like the HIV drug Maraviroc in CCR5 (Tan et al., 2013). In the CCR7 structure much of the homologous pocket surface is occupied by a desirable mix of hydrophobic and polar residues as interaction points for small molecules with an average conservation 58% between human chemokine receptors. Of particular interest for the design of orthosteric CCR7 ligands are Trp114^2.60^ and Tyr136^3.32^ where the corresponding residue is highly conserved and is directly interacting with a ligand in all known structures. Tyr312^7.38^, on the other hand, is a ligand-interacting glutamate in CCR2, CXCR4 and CCR5 but hydrogen-bonding to Tyr136^3.32^ in case of CCR7. Finding and exploring such variations in the orthosteric binding pocket will help to optimize selective ligands to pharmaceutically target CCR7 once initial hits have been identified.

Increasing the hydrophilic surface area by using fusion proteins (Chun et al., 2012) such as T4 lysozyme (Cherezov et al., 2007, Rosenbaum et al., 2007) is one of the most successful crystallization strategies for GPCRs. Because T4 lysozyme fusions failed to produce crystals in this study and to extend the toolbox for membrane protein crystallization, we identified 17 soluble proteins with optimal properties to facilitate crystallization of CCR7 (Figure S1). Out of five novel fusion constructs expressing at high amounts, three yielded crystals. The construct with the largest fusion protein Sialidase NanA (52.8 kDa) resulted in the most promising diffraction patterns. Further optimization allowed us to collect X-ray crystallographic data to a resolution up to 2.1 Å (Table S1). We employed molecular replacement combined with native single-wavelength anomalous dispersion (SAD) data obtained in a serial-scanning approach (Huang et al., 2018) to determine the structure of the human CCR7 (Figures 1, S2, and S3). Superposition of the CCR7 seven transmembrane helical bundle with that of other human chemokine receptors (CCR2 [Zheng et al., 2016], CCR5 [Tan et al., 2013], CCR9 [Oswald et al., 2016], and CXCR4 [Wu et al., 2010]) yields Cα root mean square deviations between 1.28 Å and 1.97 Å in accordance with sequence identities between 29.9% and 39.1% (Figure S4). Sequence and structural differences between the receptors are higher in the orthosteric chemokine binding pocket, on the extracellular half, than in the intracellular side, which opens upon activation to accommodate arrestin and G protein, the two major GPCR signaling proteins.

### Intracellular Allosteric Antagonism

GPCRs are intrinsically allosteric proteins that tightly couple the intracellular and extracellular halves of their 7TM bundle to fulfill their function in cellular signaling (Thal et al., 2018). Such a cooperative effect is highly desirable for cancer therapies targeting CCR7, given that it allows for more opportunities to inactivate the receptor to prevent trafficking by chemokines and thus block metastasis through the lymphatic system.

**Figure S5:**
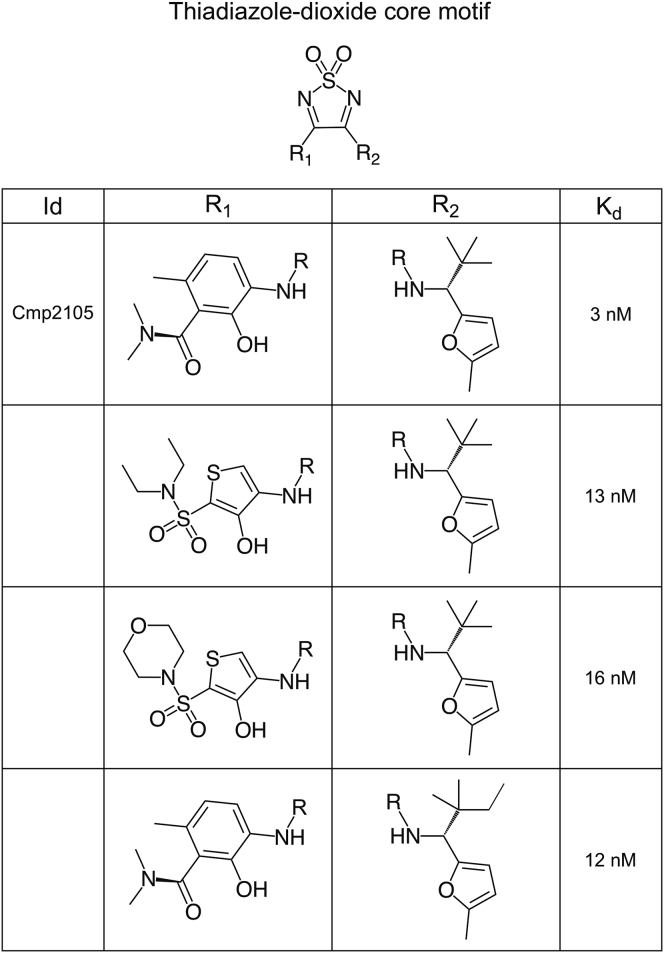
Related to Figure 2 and 3 Cmp2105 belongs to a set of four CCR7 binding molecules orginating from a large group of thiadiazole-dioxides developed and patented as potent ligands for chemokine receptors. They contain a thiadiazole-dioxide core motif with a characteristic sulfonyl group. Modification of the two amine-linked exit vectors of the central motif allows to fine-tune binding toward CCR7 (dissociation constants [K_d_] taken from Taveras et al., 2010).

**Figure 2:**
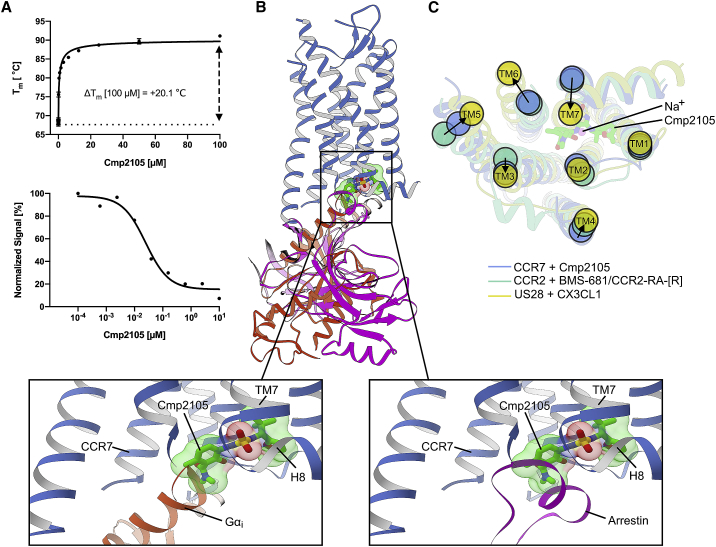
Cmp2105 Exerts Intracellular Allosteric Inhibition of CCR7 (A) Thermal-shift assays (top) verify binding of Cmp2105 to CCR7. Increasing concentrations of Cmp2105 reveal a strong dose-dependent stabilizing effect on CCR7 of up to 20.1°C (mean ± SEM from 3 independent experiments with 3 measurements each). Cmp2105 allosterically inhibits binding of the native chemokine CCL19 ligand in scintillation proximity assays (bottom) with a half inhibitory concentration (IC_50_) of 35 nM. (B) Overlay of the CCR7 structure with the position of the Gα_*i*_ subunit (Kang et al., 2018) (red) or arrestin (Kang et al., 2015) (purple) in structures of rhodopsin signaling complexes. The comparison places Cmp2105 (green; sticks and spheres) in a position where it interferes with binding of these GPCR effector proteins. (C) A structural comparison with the inactive conformation of CCR2 (Zheng et al., 2016) and the active conformation of the viral US28 with bound chemokine (Burg et al., 2015) suggests Cmp2105 to stabilize an inactive CCR7 conformation with closed intracellular effector binding site. View from the cytoplasmic side with arrows indicating relative positions in the inactive and active GPCR conformation. Our assignment to a deactivated CCR7 is further confirmed by a putative sodium ion in a conserved site between TM2, TM3, TM6, and TM7, which is known to negatively modulate activity in many GPCRs (Liu et al., 2012). Our results thus show how Cmp2105 exerts allosteric antagonism close to the intracellular G protein binding pocket of CCR7.

To date, very few small molecule ligands for CCR7 have been described. However, a large group of patented thiadiazole-dioxides contain four very similar small molecule compounds that bind CCR7 with nanomolar affinity (Taveras et al., 2010) (Figure S5). We purified and crystallized CCR7 in the presence of the thiadiazole-dioxide ligand Cmp2105. The addition of this small molecule ligand results in a strong stabilizing effect of up to 20.1°C on CCR7 (Figure 2A) in thermofluor experiments. Such a strong stabilizing effect is not untypical for a high-affinity binder. For example, the cellular entry inhibitor Maraviroc exerts a similar stabilizing effect of up to 18.9°C on the related CCR5 receptor (Knepp et al., 2011). Accordingly, we measured a half maximal inhibitory concentration (IC_50_) of 35 nM for Cmp215 in membrane-based competition experiments with radioactively labeled CCL19. Because Cmp2105 outcompeted the native protein ligand CCL19 and was classified as an antagonist to G protein activation (Taveras et al., 2010), we initially expected it to bind the orthosteric chemokine binding pocket. However, our structure showed Cmp2105 bound to a pocket at the intracellular part of CCR7 between the ends of TM1, TM2, TM3, and TM6 and the loop between TM7 and H8; this was similar to what was seen for CCR2-RA-[*R*] in CCR2 (Zheng et al., 2016), Vercirnon in CCR9 (Oswald et al., 2016), and Cmpd-15PA in the β2-adrenergic receptor (Liu et al., 2017). Cmp2105 stabilizes an inactive CCR7 conformation as evidenced by comparisons of our structure with the activated conformation of the CX3CL1-chemokine-bound viral US28 receptor (Burg et al., 2015) and the inactive conformation of the CCR2 receptor, with orthosteric and allosteric antagonists (Zheng et al., 2016) (Figure 2). Based on an overlay with the rhodopsin-Gi protein complex (Kang et al., 2018) and the rhodopsin-arrestin complex (Kang et al., 2015), the intracellular binding pocket spatially overlaps with the intracellular effector binding site of CCR7. Together with the inhibitory effect on CCL19 binding, the structural comparisons identify Cmp2105 as an intracellular allosteric antagonist. The binding site thus represents an attractive opportunity to silence CCR7 with small molecule ligands.

### Cmp2105 Binding Mode

**Figure 3:**
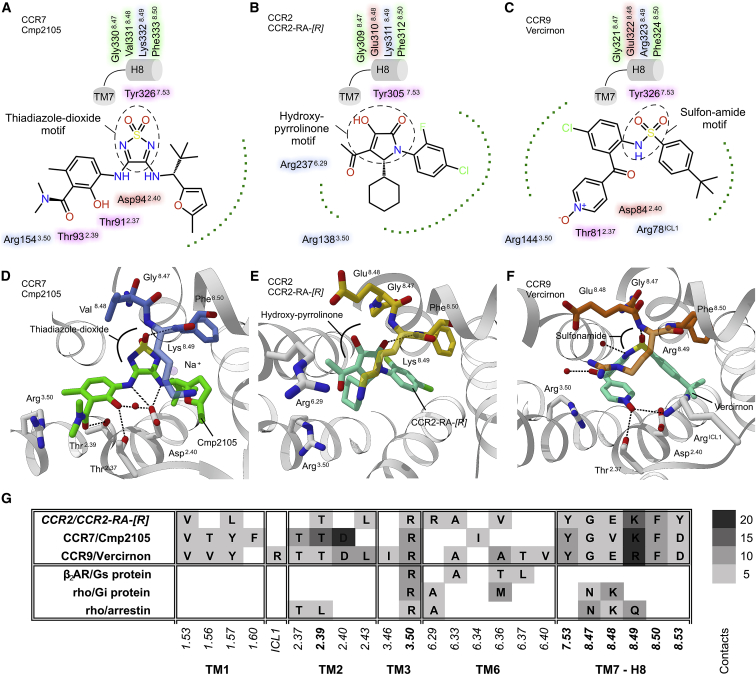
Binding Mode Comparison for Cmp2105 (CCR7), CCR2-RA-[*R*] (CCR2), and Vercirnon (CCR9) (A–C) Schematic ligand interaction profiles with protein residues colored according to their chemical nature (hydrophobic in green, polar in pink, acidic in red, and basic in blue). Dotted green lines represent regions with hydrophobic interactions. (D–F) Structural comparison of the CCR7 allosteric binding pocket with those of CCR2 and CCR9 reveals strong similarities in allosteric chemokine receptor ligand binding to a conserved patch of residues in the TM7-H8 turn (CCR7 in blue, CCR2 in yellow, and CCR9 in orange). (G) Summary of interatomic contacts (defined as pair of atoms with < 4 Å distance) in the allosteric binding pocket with CCR2-RA-[*R*] (PDB: 5T1A), Vercirnon (PDB: 5LWE), and a selection of GPCR-effector complexes (PDB: 3SN6, 6CMO, and 5DGY). Residues are shown in single-letter code with critical sites emphasized with bold letters. The grayscale indicates the number of contacts to the respective ligand or effector protein.

Cmp2105 is composed of a thiadiazole-dioxide core motif with two amine-linked substituents that can be exchanged to modulate binding affinity to CCR7 (Taveras et al., 2010) (Figure S5). The substituents form interactions to several residues in TM2 (including hydrogen bonds to Thr91^2.37^ and Thr93^2.39^) and TM1 (mainly hydrophobic Val79^1.53^, Thr82^1.56^, and Phe86^1.60^). They further bridge well-conserved residues including Arg154^3.50^ of the ERY motif in TM3 and Tyr326^7.53^ of the NPxxY motif in TM7 (Figures 3A and 3D; Table S2), both part of the cytoplasmic cleft that opens upon GPCR activation (Scheerer et al., 2008). The partial overlap with the G protein binding site, in addition to the Cmp2105 receptor interactions, hinder large conformational changes that are required for receptor activation.

The two amino groups of Cmp2105 form hydrogen bonds with Asp94^2.40^ indicating a central rolein positioning the central core motif. The sulfonyl group interacts with a conserved patch of residues at the turn between TM7 and H8 (Tyr326^7.53^, Gly330^8.47^, Val331^8.48^, Lys332^8.49^, and Phe333^8.50^). Gly330^8.47^ at the very end of TM8 is conserved among most human chemokine receptors (except CXCR6 and CXCR7) and allows the tight interhelical joint of the pocket to form. Mutations of residues Tyr^7.53^ and Phe^8.50^ in rhodopsin substantially reduce binding to Gαt (Fritze et al., 2003), and the recent cryo-EM structures of Gi/o-receptor complexes (Draper-Joyce et al., 2018, García-Nafría et al., 2018, Kang et al., 2018, Koehl et al., 2018) show how G protein selectivity is mediated by how their C termini approach the conserved motifs within the intracellular cleft including selective interactions with TM7-H8 (Tsai et al., 2018). In the structure of CCR9 (Oswald et al., 2016), the TM7-H8 motif binds a sulfonamide group of the ligand Vercirnon (Figures 3C and 3F), whereas the hydroxy-pyrrolinone ring of CCR2-RA-[*R*] occupies the equivalent position in CCR2 (Zheng et al., 2016) (Figures 3B and 3E). The conserved TM7-H8 patch (Figure 3G) within the allosteric binding pocket thus seems a promising hotspot for targeting Gαi binding chemokine receptors in agreement with the large variety of potent molecules in the thiadiazole-dioxide series of CXC- and CC-chemokine receptor ligands.

### Modulating Allosteric Ligand Recognition

**Figure 4:**
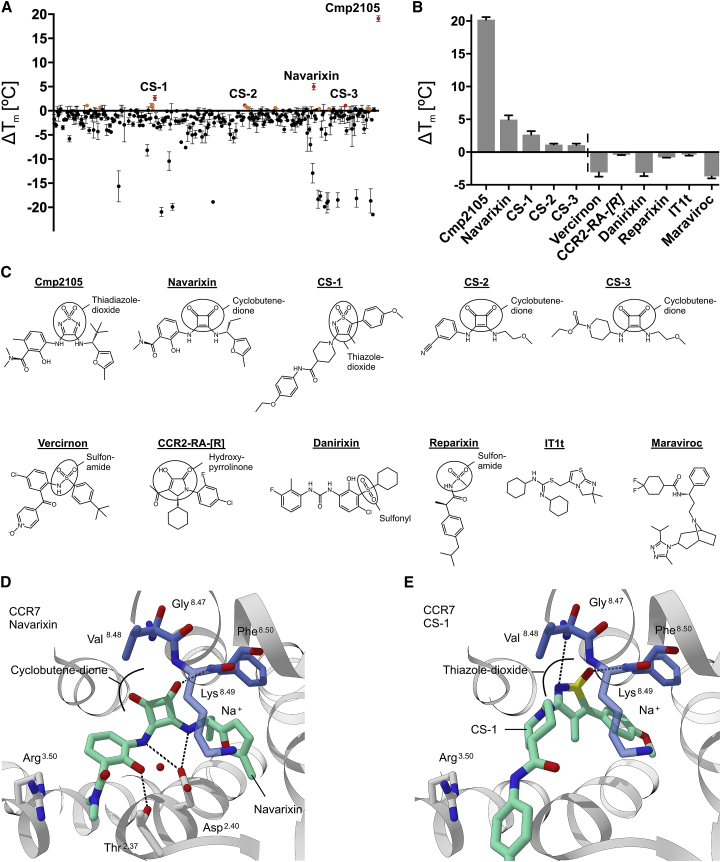
Focused Screening and Structural Diversity of Chemokine Receptor Ligands (A) 293 compounds were selected by virtual screening from Roche’s compound repository and tested experimentally for their ability to thermally stabilize CCR7. The graph plots the difference in thermal stability in presence of 50 μM ligand (black dots mean ± SEM from 3 determinations) to the DMSO control. Potentially stabilizing ligands are colored orange and hits above the combined standard deviation of DMSO and ligand are red. (B) To probe the selectivity of the binding pocket, we tested and analyzed a series of known chemokine receptor ligands and positive hits from virtual and semi-automated thermofluor screening (mean ± SEM from 3 measurements) (Mattle et al., 2018). The dashed vertical line divides measurements from the automated thermofluor assay and separate measurements with selected ligands. (C) Selection of allosteric small molecule ligands against chemokine receptors tested for their effect on CCR7. The central core motif binding the TM7-H8 motif is highlighted. (D and E) Docking of Navarixin and CS-1 into the allosteric CCR7 binding pocket. The TM7-H8 joint is shown in blue and selected key residues are drawn as sticks.

The similarity in how chemokine receptor ligands approach the Gi protein binding site in CCR2, CCR7, and CCR9 is striking. All ligands interact with the conserved TM7-H8 motif in their respective receptors. Nevertheless, CCR2-RA-[*R*] and Vercirnon did not have a thermo-stabilizing effect on CCR7, despite their similar interactions to the TM7-H8 motif in other receptors. Neither did the CXCR2 antagonist Danirixin nor the CXCR1 and CXCR2 antagonist Reparixin stabilize CCR7, although both molecules contain the characteristic sulfonamide group found in a number of chemokine ligands. Moreover, orthosteric ligands such as the CCR5 antagonist Maraviroc and the CXCR4 antagonist IT1t did not affect CCR7 (Figure 4B). This raises the question of what defines selectivity for allosteric ligands in chemokine receptors?

To address this question, we conducted a 3D shape similarity search among the 2.3 million compounds of the Roche repository. We used Cmp2105 as a seed in a focused screen to identify ligands with different topologies but a similar 3D pharmacophore. A selection of 293 fitting compounds were choosen for thermal stability assays (Figure 4A) via the automated methodology we developed originally to identify pharmacological chaperones binding to rhodopsin (Mattle et al., 2018). This combined approach revealed a series of potentially stabilizing molecules above the standard deviation of the dimethyl sulfoxide (DMSO) control, from which we selected the strongest two for further investigation. Molecule CS-1 (Figure 4C) contains the characteristic cyclic sulfone; however, the exit vectors are no longer adjacent and lack the secondary amines found in Cmp2105. Molecular docking places it into the allosteric binding pocket with the sulfonyl group facing the TM7-H8 joint (Figure 4D). No hydrogen bonds are available for interaction with Asp94^2.40^ or other nearby residues. This explains the smaller stabilizing effect of 2.4°C of this ligand but also demonstrates the importance of the interaction to the TM7-H8 core motif to approach the receptor.

**Figure 5:**
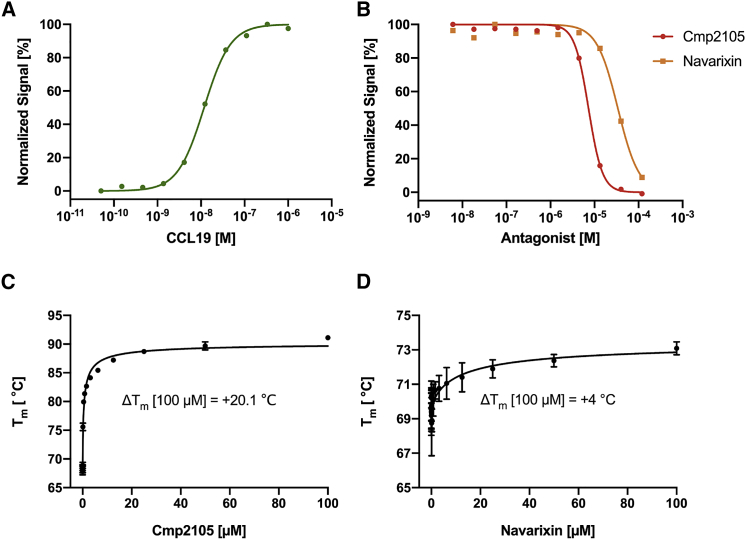
Cellular Arrestin Recruitment Assay and Dose Response Thermofluor Assay (A) Dose response curves with the native agonist CCL19 (green, circles) show arrestin binding with a half effective concentration (EC_50_) of 0.012 μM. (B) Addition of the allosteric inhibitor Cmp2105 (red, circles) or Navarixin (orange, squares) suppresses arrestin binding in response to activation by CCL19 with half inhibitory concentrations (IC_50_) of 7.3 μM and 33.9 μM respectively. Data points are the mean of two independent measurements and are normalized to the maximal response. (C) Dose-dependent thermo-stabilizing effect of Cmp2105 on CCR7. Results from 3 independent experiemnts with 3 measurements each (mean ± SEM) were fitted to extract the maximal stabilizing effect and half effective concentration of 0.48 uM for Cmp2105. (D) Dose-dependent thermo-stabilizing effect of Navarixin on CCR7. Results from three independent experiments with three measurements each (mean ± SEM) were fitted to extract the maximal stabilizing effect and half effective concentrations of 13.38 μM for Navarixin.

The second hit is of special interest because it is known as Navarixin (or SCH-527123 or MK-7123), a potent and bioavailable antagonist for CXCR1 and CXCR2 (Dwyer et al., 2006). The left- and right-hand substituents are almost identical to Cmp2105, but the central thiadiazole-dioxide core of Navarixin is replaced by a cyclobutene-dione. Molecular docking experiments place Navarixin into a similar position as Cmp2105, where it interacts with the TM7-H8 motif as well as with Asp94^2.40^. The only significant difference is the interaction of the dione in Navarixin versus the sulfonyl group in Cmp2105 (Figure 4E). The second carbonyl group of Navarixin cannot form a hydrogen bond with the protein. This could explain why the overall stabilizing effect is lower in thermal stability experiments (4°C compared with 20.1°C) and the half maximal inhibitory concentration (IC_50_) in cellular CCL19 competition assays is reduced (33.9 μM for Navarixin in comparison with 7.3 μM for Cmp2105) (Figures 5A and 5B). Overall, docking results agree well both with dose-response thermal-shift (Figures 5C and 5D) assays and the allosteric modulation of signaling in a cellular environment. Navarixin is thus validated as a biologically active allosteric antagonist for CCR7.

Clearly this part of the allosteric pocket can be targeted with a large chemical variety of small molecules to prevent binding of intracellular effector proteins. The selectivity of ligands can be tuned by changing the core motif by using a thiadiazole-dioxide (CCR7-Cmp2105), thiazole-dioxide (CCR7-CS-1), a cyclobutene-dione (CXCR1,CXCR2, and CCR7-Navarixin), a hydroxy-pyrrolinone (CCR2-CCR2-RA-[*R*]), or just the sulfonamide group alone (CCR9-Vercirnon). Once binding to the receptor with a chosen core-motif is established, near endless variations of the ligands can be achieved by modifying the substituents to fine-tune selectivity to a single or a subset of chemokine receptors.

## Conclusions

Due to their implications with cancer metastasis and their roles in the pathogenesis of autoimmune diseases, inflammation, and viral infections, chemokine receptors are important targets for pharmaceutical intervention. Despite considerable efforts, only two small molecule drugs targeting chemokine receptors are on the market today: Plerixafor (CXCR4) for stem-cell mobilization (Steinberg and Silva, 2010) and Maraviroc (CCR5) for HIV infection (Woollard and Kanmogne, 2015). Clinical failures for drugs that target chemokine receptors often result from low efficacy, low selectivity, as well as redundancies in chemokine signaling, in which the inhibition of one receptor has no significant effect because of the cell’s ability to compensate for that signaling pathway. These efforts should be reignited, considering recent insights from molecular structures and the emerging concept of using allosteric ligands to modulate GPCR signaling. The well-conserved allosteric Gi protein binding pocket on the intracellular side is a particularly interesting pharmacological target and offers advantages in comparison to the orthosteric chemokine binding pocket, as ligand selectivity can be tuned to target a single subtype or a selection of chemokine receptors. The structure elucidation of CCR7 and the validation of the allosteric binding pocket opens a new avenue to a structure-based design approach for finding novel small molecule ligands to silence chemokine receptors. Our study demonstrates this approach and presents a surprising result that Navarixin binds an allosteric pocket in CCR7. Navarixin is currently tested in phase II clinical trials (http//:clinicaltrials.gov) for its anti-metastatic effect on colorectal and other aggressive cancers (Ning et al., 2012, Varney et al., 2011). Considering the role of CCR7 in metastasis, it is not difficult to imagine that part of the Navarixin anticancer effects are due to silencing CCR7 instead of it acting solely via CXCR1/CXCR2. Drugs binding multiple targets have long been flagged as undesirable, as it was assumed this inherently leads to adverse side effects. However, a multi-target drug can also be beneficial in scenarios where redundant biological pathways lead to a compensation and resistance to single-target therapies (Ramsay et al., 2018). The identification of ligands for the relatively conserved and compact allosteric binding pocket in a subset of homeostatic chemokine receptors might thus be the best approach for alleviating multifaceted maladies such as cancer.

## STAR★Methods

### Key Resources Table

**Table:** 

REAGENT or RESOURCE	SOURCE	IDENTIFIER
**Bacterial and Virus Strains**		

Bac-to-Bac baculovirus expression system	ThermoFisher	Cat#10359016
*E.coli:* MAX Efficiency™ DH10Bac Competent Cells	ThermoFisher	Cat#10361012

**Chemicals, Peptides, and Recombinant Proteins**		

Sf900-III medium	ThermoFisher	Cat#12658027
His-tagged human Rhinovirus 3C protease (HRV 3C)	Cordingley et al., 1990	N/A
7-Diethylamino-3-(4’-Maleimidylphenyl)-4-Methylcoumarin (CPM)	ThermoFisher	Cat#D346
ChemiSCREEN™ CCR7 Membrane preparations	Millipore	N/A
PVT-PEI-WGA Type B SPA beads	Perkin Elmer	Cat#RPNQ0004
human CCL19	Prospec	Cat#CHM-374
radioactively labeled human CCL19	R&D Systems	N/A
n-Dodecyl-β-D-Maltopyranoside	Anatrace	Cat#D310
Cholesteryl Hemisuccinate Tris Salt	Anatrace	Cat#CH210
TALON Superflow Metal Affinity Resin	TaKaRa	Cat#635507
NiNTA Sepharose resin	Iba lifesciences	Cat#2-3201
Cmp2105	Roche	N/A
Navarixin	MedKoo	Cat#206586
CS-1	Roche	N/A
CS-2	Roche	N/A
Polyethylene Glycol 500 MME	Molecular Dimension	Cat#MD2-100-66
Monoolein	Nu-Check Prep	Cat#M-239
Ammonium tartrate dibasic	Sigma-Aldrich	Cat#09985
Magnesium Chloride Hexahydrate	Sigma-Aldrich	Cat#M9272
Potassium Chloride	VWR	Cat#26764.298
HEPES	Gerbu	Cat#1009
Sodium Hydroxide	VWR	Cat#28244.295
Sodium Chloride	Fisher Chemical	Cat#10598630
MES	Gerbu	Cat#1080
Bis-tris	Gerbu	Cat#1304
Glutathione (GSH)	Sigma-Aldrich	Cat#G4251
Glutathione disulfide (GSSG)	Sigma-Aldrich	Cat#G4376
Imidazole	Merck	Cat#814223
Calcium chloride dihydrate	Acros Organics	Cat#207780010
Bovine Serum Albumin Fraction V	Sigma-Aldrich	Cat#10735086001
cOmplete™ Protease Inhibitor Cocktail	Sigma-Aldrich	Cat#11697498001

**Critical Commercial Assays**		

PathHunter eXpress CCR7 CHO-K1 β-Arrestin Assay	Eurofins	Cat#93-0195E2CP0M
cAMP Hunter eXpress CCR7 CHO-K1 GPCR Assay	Eurofins	Cat#95-0070E2CP2S

**Deposited Data**		

CCR7 Crystal Structure	This manuscript	PDB: 6QZH

**Experimental Models: Cell Lines**		

*Spodoptera frugiperda Sf*9 cells	Invitrogen	Cat#11496-015

**Oligonucleotides**		

Primer pUC/M13 Forward: CCCAGTCACGACGTTGTAAAACG	Microsynth	N/A
Primer pUC/M13 Reverse: AGCGGATAACAATTTCACACAGG	Microsynth	N/A

**Recombinant DNA**		

CCR7-Sialidase construct	This manuscript	N/A

**Software and Algorithms**		

COOT	Emsley and Cowtan, 2004	N/A
XDS	Kabsch, 2010	N/A
Phaser	McCoy et al., 2007	N/A
Phenix	Adams et al., 2002	N/A
Pipeline Pilot	Dassault Systèmes BIOVIA	www.3dsbiovia.com
FastROCS	OpenEye Scientific Software	www.eyesopen.com
GOLD	CCDC, Jones et al., 1997	N/A
Prism	GraphPad	www.graphpad.com
UCSF Chimera	Pettersen et al., 2004	N/A
LigPlot+	Laskowski and Swindells, 2011	N/A

**Other**		

Laminex sandwich glass or plastic plates	Laminex, Molecular Dimensions	MD11-50-100
20 l Cell-bag Disposal Bioreactors	Wave Biotech/GE life sciences	Cat#CB0020L10-01
Mosquito LCP dispensing robot	TTP Labtech	https://www.ttplabtech.com/products/liquid-handling/mosquito-lcp/
Hamilton syringes 100 μl	Hamilton	Cat#81065
MiTeGen micromounts	MiTeGen	Cat#M2-L18SP
PD10 desalting column	GE Healthcare	Cat#17085101
Vivaspin 20, 100.000 MWCO PES	Sartorius	Cat#VS2041

### Lead Contact and Materials Availability

Further information and requests for reagents should be directed to and will be fulfilled by the Lead Contact Joerg Standfuss (joerg.standfuss@psi.ch).

### Experimental Model and Subject Details

The Bac-to-Bac baculovirus expression system (Invitrogen) was used to generate high-titer recombinant baculovirus. *Sf*9 cells at a density of 2 × 10^6^ cells/mL in SF-4 Baculo Express ICM medium (BioConcept) were infected at a multiplicity of infection of 0.01%–5% (v/v) depending on the virus strength. The cells were shaken in culture flasks (800 mL per 2 L Erlenmeyer flask) for 72 h at 27°C and 120 rpm. The cell pellet was harvested by centrifugation (3000 × g, 20 min, 4°C) and stored at −80°C.

Cellular CCR7 G protein activation assays were performed using the cAMP Hunter CHO-K1 CCR7 G_i_ Cell Line (Eurofins). Cellular CCR7 arrestin recruitment assays were done using the PathHunter® eXpress CCR7 CHO-K1 β-Arrestin GPCR Assay (Eurofins). Both assays were performed by Eurofins using standard protocols and relying on CCL19 as activating agonist.

### Methods Details

#### CCR7 Constructs and Expression

The wild type human CCR7 DNA sequence was optimized for insect cell expression and cloned into a pFastBac vector (Invitrogen). The receptor sequence (residues 1–348) was fused with enhanced green fluorescent protein (Cormack et al., 1996) for monitoring expression, followed by a C-terminal decahistidine-tag for purification. A tryptophan point mutation (L145W) was introduced to improve the thermal stability of the receptor (Roth et al., 2008). In the crystallization construct, two cleavage sites for human Rhinovirus 3C protease (Cordingley et al., 1990) have been introduced to cut the N- and C terminus between residue 36-43 and 352-359, respectively. In addition, residues Arg248^5.63^-Phe256^ICL3^ have been deleted to introduce residues 1–470 of Sialidase NanA fusion protein (PDB: 2ya4) flanked by a linker (Ser248-Lys-Leu-His-IP9-Ser-Lys-Gly-His256) on each side (Figure S1).

The CCR7 was expressed in 10 l scale in 20 l Cell-bag Disposal Bioreactors (Wave Biotech/GE life sciences) at 27°C and 19 rocks/min in a 40% oxygen atmosphere. Typically, 2 × 10^6^ Sf9 cells/mL Sf900-III medium (Invitrogen) at a viability of > 97% and an average diameter of 17–17.5 μm were infected with a volume of infection (VOI) of 0.1%–5% depending on the virus strength. The protein production was aborted at cell counts of 3–3.5 × 10^6^ cells/mL, a cell viability of ∼80% and an average diameter of 20.5–21.5 μm. This led to a fermentation of about 72 h. Cells were pelleted and then stored at −80°C until purification.

#### Purification of Sf9-Expressed CCR7 Constructs for Crystallization

All purification steps were performed at 4°C unless stated otherwise. For cell lysis, frozen cell pellets were thawed in a hypotonic buffer containing 10 mM HEPES/NaOH pH 7.5, 10 mM MgCl_2_, 20 mM KCl, Complete protease inhibitor (Roche) (1 tablet/100 mL) while stirring at 300 rpm. Cell membranes were disrupted by dounce homogenization and isolated using ultracentrifugation (234788 x g, 45 min, 4°C, Beckmann Optima-80 XE-100, rotor Ti45). Extensive washing of the membranes was performed by repeated resuspension and centrifugation with low salt buffer (10 mM HEPES/NaOH pH 7.5, 10 mM MgCl_2_, 20 mM KCl, Roche protease inhibitor tablets (1/100 mL) (1x), a high osmotic high salt buffer (10 mM HEPES/NaOH pH 7.5, 10 mM MgCl_2_, 20 mM KCl, 1 M NaCl) (2x) and low salt buffer (1x) to remove the high salt content. Purified membranes were resuspended in low salt buffer to a concentration of 1 g/mL, flash frozen in liquid nitrogen and stored at −80°C until further use.

At the purification day, membranes were thawed in water at room temperature and treated with 23 μM Cmp2105, 2 mg/mL iodoacetamide and Complete protease inhibitor (Roche) (1 tablet/50 mL). The membranes were incubated at 4°C for 1 h and solubilized in 50 mM HEPES/NaOH pH 7.5, 300 mM NaCl, 20 mM imidazole/HCl pH 7.5, 1% (w/v) DDM, 0.2% (w/v) CHS, 23 μM Cmp2105 and Complete protease inhibitor (1 tablet/50 mL) keeping a 1:1.75 ratio (amount of biomass to final volume). The mixture was stirred for 1 h at 4°C and 600 rpm and the supernatant was isolated by ultracentrifugation (234788 x g, 1 h, 4°C, Beckmann Optima-80 XE-100, rotor Ti45). The supernatant containing the solubilized receptor was incubated with pre-equilibrated Talon Superflow resin (TaKaRa, approximately 0.3 mL of resin was used for 1 g original cell pellet) and the mixture was stirred for 60 min at 4°C and 600 rpm. After binding, the resin was poured into a Falcon tube, centrifuged (500 x g, 2 min, 4°C, Eppendorf 5801 R) and washed with 10 × 2 column volumes (CV) wash buffer (50 mM HEPES/NaOH pH 7.5, 300 mM NaCl, 20 mM imidazole/HCl pH 7.5, 0.03/0.006% (w/v) DDM/CHS, 2 μM Cmp2105) in batch mode. The resin was packed into a XK16 column (GE Healthcare) column connected to a FPLC system (ÄKTA Prime, GE Healthcare) in a cooling cabinet. The resin was washed with 1 CV 20 mM imidazole and 1 CV 40 mM imidazole and then eluted in 1.5 CV 50 mM HEPES/NaOH pH 7.5, 300 mM NaCl, 200 mM imidazole/HCl pH 7.5, 0.03/0.006% (w/v) DDM/CHS, 2 μM Cmp2105. The eluted receptor was incubated with His-tagged human Rhinovirus 3C protease (HRV 3C) (in-house, 1:10 molar ratio protease:receptor) overnight in order to cleave off the N terminus and C-terminal GFP fusion and decahistidine-tag. The receptor was concentrated to a volume of < 2.5 mL using a 100 kDa molecular weight cut-off Vivaspin concentrator (Sartorius) and subsequently exchanged into a buffer containing 25 mM HEPES/NaOH pH 7.5, 150 mM NaCl, 0.03/0.006% (w/v) DDM/CHS, 2 μM Cmp2105 using a PD10 desalting column (GE Healthcare). The receptor was further purified by removing the C-terminal His-tagged GFP and the 3C HRV protease using NiNTA Sepharose resin (Iba lifesciences, 1 mL of resin per 12 mg of receptor). The pure receptor was collected as the Ni-NTA column flow-through and concentrated to 25 mg/mL. In between, the receptor was supplemented with Cmp2105 to a final concentration of 100 μM when it had reached a volume of about 1 mL. The pure, highly concentrated receptor was flash frozen in liquid nitrogen and stored at −80°C.

#### Lipidic Cubic Phase Crystallization

Prior to crystallization, the receptor was treated with 2 mM Cmp2105, incubated for 30 min on ice and reconstituted into lipidic cubic phase (LCP) using premixed molten monoolein (90% v/v)/ cholesterol (10% v/v) and two coupled Hamilton syringes. The lipid reconstitution and following steps were performed at room temperature (19-22°C). The final mixture contained 40% (v/v) protein solution, 54% (v/v) monoolein, and 6% (v/v) cholesterol. Crystallization trials were set up as 15 nL of protein-laden LCP and 800 nL of precipitant solution per well in 96-well Laminex sandwich glass or plastic plates (Laminex, Molecular Dimensions) using a Mosquito LCP dispensing robot (TTP Labtech) with a humidity chamber set to 70% humidity. Plates were incubated and imaged at 20°C using an automated imager (RockImager 1000, Formulatrix).

Diffraction quality crystals grew in 23%–27% (v/v) PEG 500 MME, 100 mM ammonium tartrate dibasic, 200 mM MES pH 6.0 or 100 mM Bis-tris pH 5.7, and with the addition of either 2.5 mM GSH (glutathione), 1-2.5 mM GSSG (glutathione disulfide) or 1-5 mM GSH/GSSG. Crystals of near cubic shape usually grew to a maximum size of 10-15 × 10-20 × 10-25 μm^3^ within 7-10 days at 20°C. Crystals were harvested from the LCP matrix using MiTeGen micromounts and flash frozen in liquid nitrogen ready for data collection.

#### Data Collection, Processing & Structure Determination

Crystallographic data were collected at the X06SA (PXI) beamline at the Swiss Light Source using a 5 × 5 μm^2^ collimated X-ray beam. Rastering for crystal centering was done using 0.03 s exposure, 90% transmission, 0.0° oscillation and a detector distance of 300 mm. Individual crystals were centered for data collection and data were collected with an exposure time of 0.025 s at 40% transmission with 0.05° oscillation and a detector distance of 300 mm. Data from 11 crystals were processed and integrated with XDS (Kabsch, 2010) and subject to STARANISO (http://staraniso.globalphasing.org/cgi-bin/staraniso.cgi) analysis for scaling and merging of the data. Data anisotropy appears to be a direct consequence of the rigid insertion protein packing along the a- and b- axes and the conformationally less restrained receptor, packing along the c-axis (Figure S2). Phaser (McCoy et al., 2007) was used to find the initial phase information using a homology model of CCR7 and a structure of the Sialidase NanA fusion protein (pdb code: 2YA4) as separate search models. The electron density was readily interpreted for the complete Sialidase NanA fusion, whereas parts of the linker and CCR7 (N-terminal residues 1-53, ICL2 residues 160-164 and ECL3 residues 289-298) were disordered. The model was refined with Phenix (Adams et al., 2002), using Rosetta refinement (DiMaio et al., 2013) in initial stages, followed by visual examination and rebuilding of the refined coordinates in COOT (Emsley and Cowtan, 2004) using anomalous differences as a guideline for placing the CCR7 sequence.

#### Virtual Screening and Molecular Docking

Using Cmp2105 as a seed compound, a substructure and 2D similarity search within the Roche compound repository was performed in Pipeline Pilot (Dassault Systèmes BIOVIA) with the extended connectivity fingerprints ECFP-6 and a Tanimoto threshold of 0.4. In addition, a 3D shape similarity search with FastROCS was carried out (OpenEye Scientific Software, Santa Fe, NM, USA. http://www.eyesopen.com). After experimental verification using thermofluor assays (see below) docking experiments were performed with the software GOLD (Jones et al., 1997) from CCDC with default settings. The best 10 docking poses were examined visually to select the most reasonable docking mode with respect to molecular interactions and internal conformational strain.

#### Thermofluor Stability Assays

The CCR7 constructs used for thermofluor stability assays were based on CCR7-L145W without the fusion protein and were expressed and purified as described above. For the generation of dose-response curves, 59 μL of purified CCR7-L145W apo-receptor at a concentration of 0.524 μM were distributed into the wells of a 96 well PCR plate on ice and 1.51 μL of a compound stock solution (at 0.1 μM – 2 mM) was added to each well, resulting in a final concentration of 0.0025 – 200 μM. The plate was sealed and incubated for 30 min on ice. A 1:100 (v/v) working solution of the CPM (N-[4-(7-diethylamino-4-methyl-3-coumarinyl)phenyl]maleimide) dye stock (3 mg/mL in DMSO) was prepared and 5.14 μL of this solution were added to each well and mixed thoroughly. Out of each well, 3 × 20 μL were distributed into 0.1 mL Rotor-Gene Q tubes (QIAGEN). The melting profiles were recorded using a real-time PCR machine (Rotor-Gene Q, QIAGEN) with temperature ramping from 25°C to 95°C in 1°C steps, 4 s pause after each step, an excitation of 365 ± 20 nm and emission of 460 ± 20 nm. The gain set was determined at the beginning of the run. The melting temperature (T_m_) was calculated from the point of inflection.

The automated thermofluor assay was performed as described (Mattle et al., 2018) with 0.54 μM CCR7-L145W and 50 μM ligand selected by virtual screening (described above). Melting curves were obtained applying a temperature gradient from 25 to 95°C and a heating rate of 0.25°C/s. All liquid-handling steps were performed using a Bravo automated liquid handling platform and a 96LT head.

#### Scintillation Proximity Assay

Scintillation proximity assays (SPA) were carried out in 96 well plates (Optiplate, Perkin Elmer) using ChemiSCREEN CCR7 Membrane preparations (Millipore), PVT-PEI-WGA Type B SPA beads (Perkin Elmer) and a mixture of radioactively labeled (2200 Ci/mmole, R&D Systems or Perkin Elmer) and non-labeled human CCL19 (Prospec). Reactions took place in 50 mM TRIS, 5 mM MgCl2, 1 mM CaCl2, 50 mM NaCl, 0.1% BSA, pH 7.6 supplemented with a CCR7 cell membrane/SPA beads mix (0.5 mg/well) and serially diluted non-labeled CCL19 (0.01 nM to 30 nM final conc.) or serially diluted Cmp2105 (1 nM to 10 μM) together with 0.05 nM labeled CCL19 in all wells. Assays were incubated for 1 h at room temperature before values were read out using a top count scintillation counter.

#### Cellular Arrestin Recruitment Assay

Arrestin recruitment was measured using the PathHunter® β-Arrestin assay (contracted to Eurofins). PathHunter® eXpress CCR7 CHO-K1 β-Arrestin cells were expanded from freezer stocks. Cells were seeded in a total volume of 20 μL Assay Complete Cell Plating Reagent (Eurofins) into white walled, 384-well microplates and incubated at 37°C. For agonist dose response curves, cells were incubated with CCL19 dissolved in DMSO (final concentration 1%) for 90 min at 37°C. For the negative allosteric modulation format, cells were pre-incubated with varying concentrations of either Cmp2105 or Navarixin followed by CCL19 challenge at its EC80 concentration (0.057 μM) for 90 min at 37°C. Assay signal was generated through a single addition of 50% v/v PathHunter Detection reagent cocktail, followed by a one h incubation at room temperature. Microplates were read following signal generation with a PerkinElmer Envision™ instrument for chemiluminescent signal detection.

### Quantification and Statistical Analyses

IC_50_ values from the scintillation proximity and cellular assays were determined using GraphPad Prism version 7.0d for Mac, GraphPad Software, La Jolla, CA, USA, http//:www.graphpad.com.

Melting temperatures (T_m_) in the thermofluor stability assays were calculated and analyzed by automated scripts based on a fit to the Boltzmann equation. Details on significance levels and number of experiments are given in the corresponding figure legends.

### Data and Code availability

#### Data Resources

The accession number for the data reported in this paper is PDB: 6QZH.

## References

[bib1] Adams P.D., Grosse-Kunstleve R.W., Hung L.W., Ioerger T.R., McCoy A.J., Moriarty N.W., Read R.J., Sacchettini J.C., Sauter N.K., Terwilliger T.C. (2002). PHENIX: building new software for automated crystallographic structure determination. Acta Crystallogr. D Biol. Crystallogr..

[bib2] Balkwill F.R. (2012). The chemokine system and cancer. J. Pathol..

[bib3] Burg J.S., Ingram J.R., Venkatakrishnan A.J., Jude K.M., Dukkipati A., Feinberg E.N., Angelini A., Waghray D., Dror R.O., Ploegh H.L., Garcia K.C. (2015). Structural biology. Structural basis for chemokine recognition and activation of a viral G protein-coupled receptor. Science.

[bib4] Cherezov V., Rosenbaum D.M., Hanson M.A., Rasmussen S.G.F., Thian F.S., Kobilka T.S., Choi H.-J., Kuhn P., Weis W.I., Kobilka B.K., Stevens R.C. (2007). High-resolution crystal structure of an engineered human beta2-adrenergic G protein-coupled receptor. Science.

[bib5] Chun E., Thompson A.A., Liu W., Roth C.B., Griffith M.T., Katritch V., Kunken J., Xu F., Cherezov V., Hanson M.A., Stevens R.C. (2012). Fusion partner toolchest for the stabilization and crystallization of G protein-coupled receptors. Structure.

[bib6] Cordingley M.G., Callahan P.L., Sardana V.V., Garsky V.M., Colonno R.J. (1990). Substrate requirements of human rhinovirus 3C protease for peptide cleavage in vitro. J. Biol. Chem..

[bib7] Cormack B.P., Valdivia R.H., Falkow S. (1996). FACS-optimized mutants of the green fluorescent protein (GFP). Gene.

[bib8] Cunningham H.D., Shannon L.A., Calloway P.A., Fassold B.C., Dunwiddie I., Vielhauer G., Zhang M., Vines C.M. (2010). Expression of the C-C chemokine receptor 7 mediates metastasis of breast cancer to the lymph nodes in mice. Transl. Oncol..

[bib9] DiMaio F., Echols N., Headd J.J., Terwilliger T.C., Adams P.D., Baker D. (2013). Improved low-resolution crystallographic refinement with Phenix and Rosetta. Nat. Methods.

[bib10] Draper-Joyce C.J., Khoshouei M., Thal D.M., Liang Y.-L., Nguyen A.T.N., Furness S.G.B., Venugopal H., Baltos J.-A., Plitzko J.M., Danev R. (2018). Structure of the adenosine-bound human adenosine A_1_ receptor-G_i_ complex. Nature.

[bib11] Dwyer M.P., Yu Y., Chao J., Aki C., Chao J., Biju P., Girijavallabhan V., Rindgen D., Bond R., Mayer-Ezel R. (2006). Discovery of 2-hydroxy-N,N-dimethyl-3-2-[[(R)-1-(5- methylfuran-2-yl)propyl]amino]-3,4-dioxocyclobut-1-enylaminobenzamide (SCH 527123): a potent, orally bioavailable CXCR2/CXCR1 receptor antagonist. J. Med. Chem..

[bib12] Emsley P., Cowtan K. (2004). Coot: model-building tools for molecular graphics. Acta Crystallogr. D Biol. Crystallogr..

[bib13] Ferlay J., Soerjomataram I., Dikshit R., Eser S., Mathers C., Rebelo M., Parkin D.M., Forman D., Bray F. (2015). Cancer incidence and mortality worldwide: sources, methods and major patterns in GLOBOCAN 2012. Int. J. Cancer.

[bib14] Förster R., Schubel A., Breitfeld D., Kremmer E., Renner-Müller I., Wolf E., Lipp M. (1999). CCR7 coordinates the primary immune response by establishing functional microenvironments in secondary lymphoid organs. Cell.

[bib15] Förster R., Davalos-Misslitz A.C., Rot A. (2008). CCR7 and its ligands: balancing immunity and tolerance. Nat. Rev. Immunol..

[bib16] Fritze O., Filipek S., Kuksa V., Palczewski K., Hofmann K.P., Ernst O.P. (2003). Role of the conserved NPxxY(x)5,6F motif in the rhodopsin ground state and during activation. Proc. Natl. Acad. Sci. USA.

[bib17] García-Nafría J., Nehmé R., Edwards P.C., Tate C.G. (2018). Cryo-EM structure of the serotonin 5-HT_1B_ receptor coupled to heterotrimeric G_o_. Nature.

[bib18] Günther K., Leier J., Henning G., Dimmler A., Weissbach R., Hohenberger W., Förster R. (2005). Prediction of lymph node metastasis in colorectal carcinoma by expression of chemokine receptor CCR7. Int. J. Cancer.

[bib19] Hauser A.S., Attwood M.M., Rask-Andersen M., Schiöth H.B., Gloriam D.E. (2017). Trends in GPCR drug discovery: new agents, targets and indications. Nat. Rev. Drug Discov..

[bib20] Horuk R. (2009). Chemokine receptor antagonists: overcoming developmental hurdles. Nat. Rev. Drug Discov..

[bib21] Huang C.Y., Olieric V., Howe N., Warshamanage R., Weinert T., Panepucci E., Vogeley L., Basu S., Diederichs K., Caffrey M., Wang M. (2018). In situ serial crystallography for rapid de novo membrane protein structure determination. Commun Biol.

[bib22] Jones G., Willett P., Glen R.C., Leach A.R., Taylor R. (1997). Development and validation of a genetic algorithm for flexible docking. J. Mol. Biol..

[bib23] Kabsch W. (2010). XDS. Acta Crystallogr. D Biol. Crystallogr..

[bib24] Kang Y., Kuybeda O., de Waal P.W., Mukherjee S., Van Eps N., Dutka P., Zhou X.E., Bartesaghi A., Erramilli S., Morizumi T. (2018). Cryo-EM structure of human rhodopsin bound to an inhibitory G protein. Nature.

[bib25] Kang Y., Zhou X.E., Gao X., He Y., Liu W., Ishchenko A., Barty A., White T.A., Yefanov O., Han G.W. (2015). Crystal structure of rhodopsin bound to arrestin by femtosecond X-ray laser. Nature.

[bib26] Knepp A.M., Grunbeck A., Banerjee S., Sakmar T.P., Huber T. (2011). Direct measurement of thermal stability of expressed CCR5 and stabilization by small molecule ligands. Biochemistry.

[bib27] Koehl A., Hu H., Maeda S., Zhang Y., Qu Q., Paggi J.M., Latorraca N.R., Hilger D., Dawson R., Matile H. (2018). Structure of the μ-opioid receptor-G_i_ protein complex. Nature.

[bib28] Laskowski R.A., Swindells M.B. (2011). LigPlot+: multiple ligand-protein interaction diagrams for drug discovery. J. Chem. Inf. Model..

[bib29] Liu W., Chun E., Thompson A.A., Chubukov P., Xu F., Katritch V., Han G.W., Roth C.B., Heitman L.H., IJzerman A.P. (2012). Structural basis for allosteric regulation of GPCRs by sodium ions. Science.

[bib30] Liu X., Ahn S., Kahsai A.W., Meng K.-C., Latorraca N.R., Pani B., Venkatakrishnan A.J., Masoudi A., Weis W.I., Dror R.O. (2017). Mechanism of intracellular allosteric β_2_AR antagonist revealed by X-ray crystal structure. Nature.

[bib31] Mattle D., Kuhn B., Aebi J., Bedoucha M., Kekilli D., Grozinger N., Alker A., Rudolph M.G., Schmid G., Schertler G.F.X. (2018). Ligand channel in pharmacologically stabilized rhodopsin. Proc. Natl. Acad. Sci. USA.

[bib32] McCoy A.J., Grosse-Kunstleve R.W., Adams P.D., Winn M.D., Storoni L.C., Read R.J. (2007). Phaser crystallographic software. J. Appl. Cryst..

[bib33] Mishan M.A., Ahmadiankia N., Bahrami A.R. (2016). CXCR4 and CCR7: Two eligible targets in targeted cancer therapy. Cell Biol. Int..

[bib34] Moschovakis G.L., Bubke A., Friedrichsen M., Ristenpart J., Back J.W., Falk C.S., Kremmer E., Förster R. (2018). The chemokine receptor CCR7 is a promising target for rheumatoid arthritis therapy. Cell. Mol. Immunol..

[bib35] Müller A., Homey B., Soto H., Ge N., Catron D., Buchanan M.E., McClanahan T., Murphy E., Yuan W., Wagner S.N. (2001). Involvement of chemokine receptors in breast cancer metastasis. Nature.

[bib36] Ning Y., Labonte M.J., Zhang W., Bohanes P.O., Gerger A., Yang D., Benhaim L., Paez D., Rosenberg D.O., Nagulapalli Venkata K.C. (2012). The CXCR2 antagonist, SCH-527123, shows antitumor activity and sensitizes cells to oxaliplatin in preclinical colon cancer models. Mol. Cancer Ther..

[bib37] Oswald C., Rappas M., Kean J., Doré A.S., Errey J.C., Bennett K., Deflorian F., Christopher J.A., Jazayeri A., Mason J.S. (2016). Intracellular allosteric antagonism of the CCR9 receptor. Nature.

[bib60] Pettersen E.F., Goddard T.D., Huang C.C., Couch G.S., Greenblatt D.M., Meng E.C., Ferrin T.E. (2004). UCSF Chimera–a visualization system for exploratory research and analysis. J Comput. Chem.

[bib38] Pron B., Boumaila C., Jaubert F., Berche P., Milon G., Geissmann F., Gaillard J.L. (2001). Dendritic cells are early cellular targets of Listeria monocytogenes after intestinal delivery and are involved in bacterial spread in the host. Cell. Microbiol..

[bib39] Qin L., Kufareva I., Holden L.G., Wang C., Zheng Y., Zhao C., Fenalti G., Wu H., Han G.W., Cherezov V. (2015). Structural biology. Crystal structure of the chemokine receptor CXCR4 in complex with a viral chemokine. Science.

[bib40] Ramsay R.R., Popovic-Nikolic M.R., Nikolic K., Uliassi E., Bolognesi M.L. (2018). A perspective on multi-target drug discovery and design for complex diseases. Clin. Transl. Med..

[bib41] Rosenbaum D.M., Cherezov V., Hanson M.A., Rasmussen S.G.F., Thian F.S., Kobilka T.S., Choi H.-J., Yao X.-J., Weis W.I., Stevens R.C., Kobilka B.K. (2007). GPCR engineering yields high-resolution structural insights into beta2-adrenergic receptor function. Science.

[bib42] Roth C.B., Hanson M.A., Stevens R.C. (2008). Stabilization of the human beta2-adrenergic receptor TM4-TM3-TM5 helix interface by mutagenesis of Glu122(3.41), a critical residue in GPCR structure. J. Mol. Biol..

[bib43] Scheerer P., Park J.H., Hildebrand P.W., Kim Y.J., Krauss N., Choe H.-W., Hofmann K.P., Ernst O.P. (2008). Crystal structure of opsin in its G-protein-interacting conformation. Nature.

[bib44] Shields J.D., Fleury M.E., Yong C., Tomei A.A., Randolph G.J., Swartz M.A. (2007). Autologous chemotaxis as a mechanism of tumor cell homing to lymphatics via interstitial flow and autocrine CCR7 signaling. Cancer Cell.

[bib45] Solari R., Pease J.E., Begg M. (2015). “Chemokine receptors as therapeutic targets: Why aren’t there more drugs?”. Eur. J. Pharmacol..

[bib46] Steinberg M., Silva M. (2010). Plerixafor: A chemokine receptor-4 antagonist for mobilization of hematopoietic stem cells for transplantation after high-dose chemotherapy for non-Hodgkin’s lymphoma or multiple myeloma. Clin. Ther..

[bib47] Tan Q., Zhu Y., Li J., Chen Z., Han G.W., Kufareva I., Li T., Ma L., Fenalti G., Li J. (2013). Structure of the CCR5 chemokine receptor-HIV entry inhibitor maraviroc complex. Science.

[bib48] Taveras, A., Chao, J., Biju, P., Yu, Y., Fine, J., Hipkin, W., Aki, C., Merritt, R., Li, G., Baldwin, J., et al. (2010). Thiadiazoledioxides and thiadiazoleoxides as cxc- and cc-chemokine receptor ligands. US patent US7691856B2. Filed January 09, 2007, and granted April 06, 2010.

[bib49] Thal D.M., Glukhova A., Sexton P.M., Christopoulos A. (2018). Structural insights into G-protein-coupled receptor allostery. Nature.

[bib50] Tsai C.-J., Pamula F., Nehmé R., Mühle J., Weinert T., Flock T., Nogly P., Edwards P.C., Carpenter B., Gruhl T. (2018). Crystal structure of rhodopsin in complex with a mini-Go sheds light on the principles of G protein selectivity. Science Advances.

[bib51] Varney M.L., Singh S., Li A., Mayer-Ezell R., Bond R., Singh R.K. (2011). Small molecule antagonists for CXCR2 and CXCR1 inhibit human colon cancer liver metastases. Cancer Lett..

[bib52] Weinert T., Olieric V., Waltersperger S., Panepucci E., Chen L., Zhang H., Zhou D., Rose J., Ebihara A., Kuramitsu S. (2015). Fast native-SAD phasing for routine macromolecular structure determination. Nat. Methods.

[bib53] Wiley H.E., Gonzalez E.B., Maki W., Wu M.T., Hwang S.T. (2001). Expression of CC chemokine receptor-7 and regional lymph node metastasis of B16 murine melanoma. J. Natl. Cancer Inst..

[bib54] Wojdyla J.A., Kaminski J.W., Panepucci E., Ebner S., Wang X., Gabadinho J., Wang M. (2018). DA+ data acquisition and analysis software at the Swiss Light Source macromolecular crystallography beamlines. J. Synchrotron Radiat..

[bib55] Wojdyla J.A., Panepucci E., Martiel I., Ebner S., Huang C.Y., Caffrey M., Bunk O., Wang M. (2016). Fast two-dimensional grid and transmission X-ray microscopy scanning methods for visualizing and characterizing protein crystals. J. Appl. Cryst..

[bib56] Woollard S.M., Kanmogne G.D. (2015). Maraviroc: a review of its use in HIV infection and beyond. Drug Des. Devel. Ther..

[bib57] Wu B., Chien E.Y.T., Mol C.D., Fenalti G., Liu W., Katritch V., Abagyan R., Brooun A., Wells P., Bi F.C. (2010). Structures of the CXCR4 chemokine GPCR with small-molecule and cyclic peptide antagonists. Science.

[bib58] Zheng Y., Qin L., Zacarías N.V.O., de Vries H., Han G.W., Gustavsson M., Dabros M., Zhao C., Cherney R.J., Carter P. (2016). Structure of CC chemokine receptor 2 with orthosteric and allosteric antagonists. Nature.

[bib59] Zlotnik A., Burkhardt A.M., Homey B. (2011). Homeostatic chemokine receptors and organ-specific metastasis. Nat. Rev. Immunol..

