# Supplementary material for: Structural Basis for Allosteric Ligand Recognition in the Human CC Chemokine Receptor 7
Source: Cell. 2019 Aug 22;178(5):1222–1230.e10. doi: 10.1016/j.cell.2019.07.028 (PMC6709783; doi:10.1016/j.cell.2019.07.028)
Supplement: Document S1. Tables S1 and S2 [file mmc1.pdf]

Cell, Volume *178*

Supplemental Information

Structural Basis for Allosteric Ligand Recognition in the Human CC Chemokine Receptor 7

Kathrin Jaeger, Steffen Bruenle, Tobias Weinert, Wolfgang Guba, Jonas Muehle, Takuya Miyazaki, Martin Weber, Antonia Furrer, Noemi Haenggi, Tim Tetaz, Chia-Ying Huang, Daniel Mattle, Jean-Marie Vonach, Alain Gast, Andreas Kuglstatter, Markus G. Rudolph, Przemyslaw Nogly, Joerg Benz, Roger J.P. Dawson, and Joerg Standfuss

**Data Statistics**	**CCR7**	**$CCR7_{6keV}$**
Crystals merged	11	726
Space group	$P2_12_12$	$P2_12_12$
Unit cell (a; b; c; α=β=γ)	79.1; 128.8; 100.6; 90.0	79.1; 129.0; 100.0; 90.0
Wavelength (Å)	1.0	2.066
Resolution (Å)	79.3 – 2.1 (2.4 – 2.1)	50.0 – 3.0 (3.1-3.0)
R_{pim}	10.0 (92.3)	7.6 (163.4)
I/σI	8.1 (1.6)	12.64 (1.6)
Spherical completeness (%)	53.3 (8.7)	100.0 (100.0)
Ellipsoidal completeness (%)	93.7 (83.0)	
Ellipsoidal truncation resolution limits along a*/b*/c*(Å)	2.66 / 2.12 / 3.06	no truncation applied
Multiplicity	27.4 (33.8)	134.5 (103.6)
$CC_{1/2}$	98.6 (63.9)	99.7 (82.9)
**Refinement Statistics**		
Resolution	54.2 – 2.1	
No. Reflections	31344	
R_{work} / R_{free}	19.6 / 24.4	
Ramachandran favored	92.3	
Ramachandran outliers	0.53	
R.m.s.d. Bond length (Å)	0.003	
R.m.s.d. Bond angles (°)	0.589	
PDB Code	6QZH	

Table S1: Related to Figure 1. X-ray crystallographic data

**Helix Loop**	**allosteric**							**orthosteric**			
		**CCR2 CCR2-RA-[*R*]**	**CCR7 Cmp2105**	**CCR9 Vercirnon**	**β2AR G_s**	**rhodopsin G_i**	**rhodopsin arrestin**		**CCR2 BMS-681**	**CXCR4 IT1t**	**CCR5 Maraviroc**
**TM1**	1.53		Val79	Val69				1.26		Glu32	
	1.56		Thr82	Val72				1.27	Va37		
	1.57		Tyr83	Tyr73				1.31	Gly41		
	1.60		Phe86					1.35	Leu45		
								1.39	***Tyr49**		
**ICL1**				Arg78							
			Leu89								
**TM2**	2.37		***Thr91**	***Thr81**			Thr70	2.60	Trp98	Trp94	Trp86
	2.39	Thr77	***Thr93**	Thr83			Leu72	2.63		***Asp97**	Tyr89
	2.40		****Asp94**	Asp84							
	2.43	Leu81		Leu87							
**ELC1**										Trp102	
**TM3**	3.50		Arg154	Arg144	Arg131	Arg135	Arg135	3.29	Thr117		
	3.53				Ala134			3.32	Tyr120	Tyr116	Tyr108
	3.54				Ile135		Val139	3.33			Phe109
	3.55				Thr136		Cys140	3.36			Phe112
	3.56						Lys141				
**ICL2**					Pro138		Pro142				
					Phe139		Met143				
					Tyr141		Asn145				
					Gln142		Phe146				
					Ser143	Arg147	Arg147				
**ELC2**										Arg183	
										Ile185	
									Cys190	Cys186	
										Asp187	
										Arg188	
**TM5**	5.61				Val222		Leu226	5.39			Gln194
	5.64				Glu225		Thr229	5.40			Thr195
	5.65				Ala226			5.43			Ile198
	5.68				Gln229		Ala233				
	5.69				Leu230						
	5.71				Lys232		Gln236				
	5.72				Ile233	Gln237	Gln237				
	5.74					Glu239	Glu239				
	5.78				Arg239						
**TM6**	6.23					Ser240					
	6.24					Ala241					
	6.25					Thr242	Thr242				
	6.28					Lys245					
	6.29	Arg237				Ala246	Ala246	6.48			Trp248
	6.32					Glu249	Glu249	6.51			***Tyr251**
	6.33	Ala241			Ala271		Val250	6.55			Leu255
	6.36	Val244		Ala255	Thr274	Met253					
	6.37		Ile265	Thr256	Leu275						
	6.40			Va259							
**TM7**	7.53	Tyr305	Tyr326	Tyr317				7.34			Met279
								7.35	***Gln288**		
								7.38	Glu291	***Glu288**	Glu283
								7.39	***Thr292**		Thr284
	7.56					Met309		7.42	Met295		
**H8**	8.47	Gly309	Gly330	Gly321		Asn310	Asn310				
	8.48	Glu310	Val331	Glu322		Lys311	Lys311				
	8.49	Lys311	****Lys332**	***Arg323**			Gln312				
	8.50	***Phe312**	***Phe333**	***Phe324**							
	8.53			Asp327							

Table S2: Related to Figure 3. Contact regions between small molecule ligands in chemokine receptors and between receptor-effector complexes. Interactions in the allosteric (left) and orthosteric (right) binding pockets in CCR2 (pdb code: 5t1a), CXCR4 (pdb code: 3odu), CCR5 (pdb code: 4mbs), CCR7 (pdb code: 6qzh) and CCR9 (pdb code: 5lwe) are listed according to the Ballesteros-Weinstein numbering system for GPCRs. Residues involved into hydrogen bonding with a small molecule ligand shown in bold and are marked based on selection using LigPlot+(Laskowski and Swindells, 2011). A selection of the Gs protein (pdb code: 3sn6), Gi protein (pdb code: 3cmo) and arrestin (pdb code: 4zwj) complexes are included as comparison in which residues were included when they had a minimum distance of 4Å between their closest atoms.
